# The Poor-Law Institutions Order: The Duties of Workhouse Medical Officers

**Published:** 1914-02-14

**Authors:** 


### THE POOR-LAW INSTITUTIONS ORDER.

### The Duties of Workhouse Medical Officers.

In view of the recent correspondence in our columns the following summary of the duties, powers, and responsibilities of workhouse medical officers under the Poor-Law Institutions Order may prove of service to those officers and of interest to others. The Order states that the medical officer must personally discharge the duties of his office, except so far as provision is made by or with the approval of the Guardians for assistance in his work, and where assistance is so provided he is to be personally responsible for the proper discharge of those duties.

A. General duties of the medical officer.

1. In all matters affecting his office he is to comply with all requirements contained in any Order of the Local Government Board; obey all lawful orders of the Guardians; make written reports when required by the Local Government Board, the Guardians, any committee of Guardians, or the Clerk; and supply to the Clerk any information required for the business of the union.

2. Attend any meeting of the Guardians or Committee of Guardians, and produce to them or to the Clerk all books, documents, or accounts kept by him or in his custody when required.

3. Account for all property of the Guardians under his charge.

4. Attend the inmates of the institution at the times fixed by the Guardians, when sent for in the manner provided by the Order, and at such other times as may be necessary, and give all necessary directions for the treatment and nursing of the sick.

5. Employ in all records the terms used in the "Nomenclature of Diseases."

B. Duties with respect to the examination of inmates.

1. Examine all inmates on admission.

2. Re-examine any inmate at his request, or in accordance with the Guardians' regulations in the matter, if the question of his transfer from one class to another arises.

3. Examine all infants under eighteen months at least fortnightly, and all infants over eighteen months and all children at least monthly.

4. Examine every child or infant within twenty-four hours before, and every lunatic immediately before, transfer from the institution.

and records, and the making of regulations, reports, and certificates.

C. Duties with respect to the keeping of books

1. Enter in a special book the result of his examination on admission, and of his re-examination of any inmate.

2. Enter in a special book the result of his examination on admission and transfer of every lunatic or alleged lunatic.

3. Keep a book showing the date and time any lunatic or alleged lunatic is compulsorily secluded or placed in the padded room.

4. Keep records (1-7) in the Alcohol Book.

5. Keep a record paper in the form prescribed in the case of each inmate of the sick or lunatic wards, and of each infant under eighteen months.—Enter on the record paper all necessary directions in respect of the feeding of each infant under eighteen months.

6. Keep a record of his periodic examinations of children and of infants over eighteen months, and of the complaint and treatment whenever an inmate applies for or is ordered medical treatment.

7. Certify in writing the result of his examination of every child and infant on transfer from the institution.

8. Give to any inmate at his request a written certificate of the cause of his attendance upon him.

9. Initial all record papers at least once every three months if there is an assistant medical officer, and as soon as practicable after an inmate's death or discharge, and all entries made by the midwife on the record paper of a maternity case at his next visit, and all requisitions for provisions or alcoholic liquors made by a nurse or other officer in his absence.

10. Report to the Local Government Board within twenty-four hours any accidental or sudden death of an inmate of the institution, unless the accident occurred at a time when the deceased was not in receipt of relief.

11. Report to the house committee any matters arising from his examination of the children and infants over eighteen months, and in a special book, as occasion may arise, upon any matter affecting the health of the inmates, the sanitation of the building, or the nursing of the sick and infants, the occurrence of any sudden or accidental death among the inmates, and any requisition he may have made upon the master for an extra nurse.

12. Give the Guardians or house committee, when required by them, any reasonable information respecting the case of any inmate.

13. Make twice a year to the house committee a report of the condition of the institution.

14. Advise the house committee and the master of any steps he may deem advisable to prevent the spread of infection.

15. Inform the master forthwith of any case of serious illness, and, immediately after the death of a lunatic or alleged lunatic, furnish him with a report, on a prescribed form, of the cause of death, a description of any injuries present at death, any unusual circumstances attending the death, and whether deceased was placed under restraint or in seclusion within seven days previous to death.

16. Furnish the medical officer of health with such information as the Local Government Board may direct with respect to cases of sickness or death within the institution.

17. Draw up on a prescribed form dietaries for the inmates of the sick and lunatic wards and for infants over eighteen months of age.

18. Draw up a code of instructions for the bathing of inmates of the sick and lunatic wards, and of infants.

### D. Cases in which the medical officer must be consulted.

1. In forming classes to include infants, persons who are infirm or suffering from disease of body or mind, or women who are pregnant, or have recently been confined, or are suckling infants; and in assigning accommodation to those classes the Guardians shall have regard to recommendations to be obtained in writing from him.

2. No room is to be used for punishing an inmate by confinement therein unless he has certified that it is fit to be so used.

3. He must certify in writing that injury to the inmate's health is not reasonably to be apprehended from the proposed punishment, before an inmate over sixty or under twelve, sick or of unsound mind, recently confined, or suckling an infant, under his care within the preceding seven days or pronounced by him to be pregnant, can be punished. Any diminution of the punishment suggested by him must be adopted by the master.

4. No infant in arms whose mother is in the institution can be weaned except on his written direction.

5. The Guardians must obtain his written advice before making regulations with regard to visits to inmates of the sick or lunatic wards or the nurseries, and before making the dietary tables.

6. If the staff of the institution does not include a trained nurse the Guardians must obtain his advice before making provision for skilled nursing attendance.

7. An inmate pregnant, recently confined, or suckling an infant is to be employed only on such work and for such hours as he may approve, and no inmate is to be employed in the sick or lunatic wards or nurseries except with his approval for the particular employment.

8. A certificate must be obtained from him before an adult inmate can receive an infirm diet or an additional meal, or the special infirm diet.

### E. Cases in which the medical officer may vary the regulations governing the institution.

1. He may postpone the transfer of a child for medical reasons by giving a certificate.

2. He may direct that an inmate on his admission shall not be bathed. He may give such directions as he may think necessary in regard to the bathing or cleansing of any inmate.

3. On his written advice the Guardians may direct an inmate for special reasons to be placed in premises set apart for the reception of inmates of another class.

4. The right of the parents of an infant or child in the same institution to visit it at least daily is subject to his directions.

5. The employment of inmates is subject to any directions given by him on the grounds of health or mental capacity.

6. In the case of the sick wards he may direct a bed-card to be fixed near the bed of an inmate instead of a record paper.

7. He may direct the diet of an inmate who is insane, pregnant, recently confined, or suckling an infant to be varied for a stated period not exceeding one month.

8. He may give similar directions in the case of any other inmate. In this case the directions must be obeyed until the next meeting of the house committee, and, if this committee approves, for the whole period stated.

9. He may give a certificate at any time that a temporary change of diet is essential to the health of any or all of the inmates, and the Guardians may then alter their dietary for a period not exceeding one month.

10. Alcoholic liquors are not to be allowed an inmate who is not in the sick or lunatic wards. The medical officer may, however, make a special recommendation that such an allowance is necessary for the health of any such inmate, and the Guardians may then order it to be given for a period not exceeding twenty-eight days.

### F. Other powers given to the medical officer.

1. An inmate found on admission to be sick or in an infectious state is to be transferred on his written advice to an appropriate ward or institution.

2. The inmates of the sick or lunatic wards and the infants are to receive the medicines and attendance, the diets, and extras ordered them by him. His entries on the record papers prescribing diet or extras will authorise their supply for a period not exceeding one month, and in the case of alcoholic liquors for a period not exceeding eight days.

3. His directions as regards the treatment of the sick and the infants, and the heating and ventilation of the sick wards and nurseries are to be observed.

4. When there is a head nurse, and the matron does not possess the qualifications prescribed for that office, the head nurse is in matters relating to the treatment and nursing of the sick to act immediately under his directions.

5. The superintendent nurse is to act under his directions in training the probationers.

6. In any case of urgency upon his written report of the need of a temporary nurse, the master is to engage one until the next meeting of the Guardians.

### G. Cases in which the medical officer must be informed.

1. Whenever the prompt examination of an inmate on his admission is necessary or the master sends him direct to a ward.

2. Whenever an inmate of the sick wards gives notice of his intention to discharge himself.

3. Whenever an inmate dies.

4. Whenever any restraint or compulsion has, in his absence, been used in the case of a lunatic or alleged lunatic.

5. Whenever any defects are observed in the wards under the supervision of the matron or superintendent nurse, or there are any matters requiring consideration therewith or with the nursing of the sick.

### H. Cases in which the medical officer must be sent for.

1. Whenever any inmate is taken ill or becomes insane, or any lunatic or alleged lunatic, any sick person or any person suspected to have been exposed to infection is admitted to the institution, in any other case of urgency, and under any circumstances in which the rules of the Central Midwives Board would require a midwife to take steps to obtain the attendance of a registered medical practitioner.

